# The role of health beliefs and health literacy in women's health promoting behaviours based on the health belief model: a descriptive study

**DOI:** 10.1186/s12905-021-01564-2

**Published:** 2021-12-18

**Authors:** Mahla Ghorbani-Dehbalaei, Marzeyeh Loripoor, Mostafa Nasirzadeh

**Affiliations:** 1 Department of Health Education and Health Promotion, School of Health, Student Research Committee, Rafsanjan University of Medical Sciences, Rafsanjan, Iran; 2 Department of Midwifery, School of Nursing and Midwifery, Geriatric Care Research Center, Rafsanjan University of Medical Sciences, Rafsanjan, Iran; 3 Department of Health Education and Health Promotion, School of Health, Occupational Safety and Health Center, NICICO, World Safety Organization and Rafsanjan University of Medical Sciences, Rafsanjan, Iran

**Keywords:** Health literacy, Gynecological diseases, Health belief model

## Abstract

**Background:**

Health literacy and health beliefs are factors that can effectively contribute to adoption of preventive behaviors among women. The present study was done to explore the role of health beliefs and health literacy in women's health promoting behaviors based on the health belief model (HBM).

**Methods:**

The descriptive study was conducted in 2020 on 431 female students of Rafsanjan University of Medical Sciences (RUMS) who had been selected through stratified sampling. Data collection tool was a questionnaire which covered eight demographic information, 41 health literacy questions and 50 researcher-developed questions of health belief based on HBM constructs. Data were collected electronically and SPSS version 20 and independent *t*-test, one-way ANOVA, Pearson correlation coefficient and Multiple Linear Regression were used for data analysis at a significance level less than 0.05.

**Results:**

The preventive behaviors were adopted by 75.57% of the population and the total health literacy score was found to be 52.71 out of 100. According to the Multiple regression analysis, self-efficacy (β = 0.414, *p* = 0.001) and cues to action (β = 0.299, *p* = 0.001) were found to be the first and second robust predictors of behavior, respectively. Health literacy, self-efficacy, cues to action and perceived susceptibility constructs predicted 52.1% of preventive behaviors.

**Conclusion:**

It is recommended that researchers design, implement and evaluate interventions based on behavioral change theories, especially the self-efficacy theory, in order to promote women's health.

**Supplementary Information:**

The online version contains supplementary material available at 10.1186/s12905-021-01564-2.

## Background

Given the biological, cultural, social, economic, and political factors, women are more vulnerable than men, and they are more exposed to health risks than men due to physical, sexual, and mental differences [1, 2]. Fertility and childbirth, as well as menstruation expose women to specific health risks including menstrual cramps, iron deficiency anemia, genital infections, sexually-transmitted diseases, preterm labor, cervical cancer, breast cancer and female mortality at young ages [2].

Anemia is the most common nutritional disorder in the world, as many as 12.2% of adolescent girls, 3.8% of young adult women in the world, and 17% of Iranian women suffer from iron deficiency anemia [3, 4]. Breast cancer is also the most common cancer among women; it accounts for 30% of all cancers in women, and it is the main cause of 15% of cancer-related deaths in women [5]. The incidence rate of breast cancer in Iranian women is 5.27 per 100,000 women [5]. Menstrual health as another important issue for women's health is an integral part of overall health, nevertheless, millions of women around the world, menstruation regularly and increasingly disrupts their physical, mental, and social well-being [6].

The results of a study conducted by Saadatmand et al. among the students of Islamic Azad University of Qom indicated that only 7.3% had desirable and good menstrual health behaviors [7].

Health literacy (H.L), as a term first introduced in the 1970s, [8] generally concerns whether an individual is competent with the complex demands of promoting and maintaining health in the modern society [9].

H.L is an important element in a woman’s ability to engage in health-promoting activities [10]. Without a good understanding of health care information, informed decisions leading to desirable health results will be difficult for a woman [11]. In their review study, Mousavi and Bagherian Sararoudi (2019) confirmed that H.L can be effective in preventing breast cancer and managing the symptoms arising from this disease [12].

Nutrition and Physical activity are the most important part of a healthy lifestyle in women that directly affects their health problems [13]. Adopting a low-fat diet, along with increasing the consumption of vegetables, fruits and whole grains, can reduce the risk of death from breast cancer in women [14]. Improper diet and nutrition patterns among young people can trigger various diseases at later ages [15]. Researchers argue that several factors are likely to affect the nutritional status of young people; these factors include gender, body weight, length of college years, avoiding certain types of valuable foods, and nutritional patterns formed before entering university [15]. Different studies have indicated that the consumption of essential nutrients such as iron, zinc, magnesium, calcium and folic acid among college students, especially female students, is less than what is recommended [16].

Moreover, numerous studies have also confirmed the benefits of regular physical activity as another important factor affecting health, since a sedentary lifestyle is associated with the risk of many chronic diseases; every year, about two million deaths are reported worldwide due to adopting a sedentary lifestyle [17]. Different studies have reported that awareness, perceived severity, and self-efficacy are the main variables predicting women’s physical activity [18, 19].

Behavior change theories and models provide a systematic view of events or successes, and they are assumed as a regular process for analyzing successes or failures, as a training process map, they provide the required guidelines for educational diagnosis and planning, and intervention design, and they facilitate evaluation as well [20, 21].

**Fig. 1:**
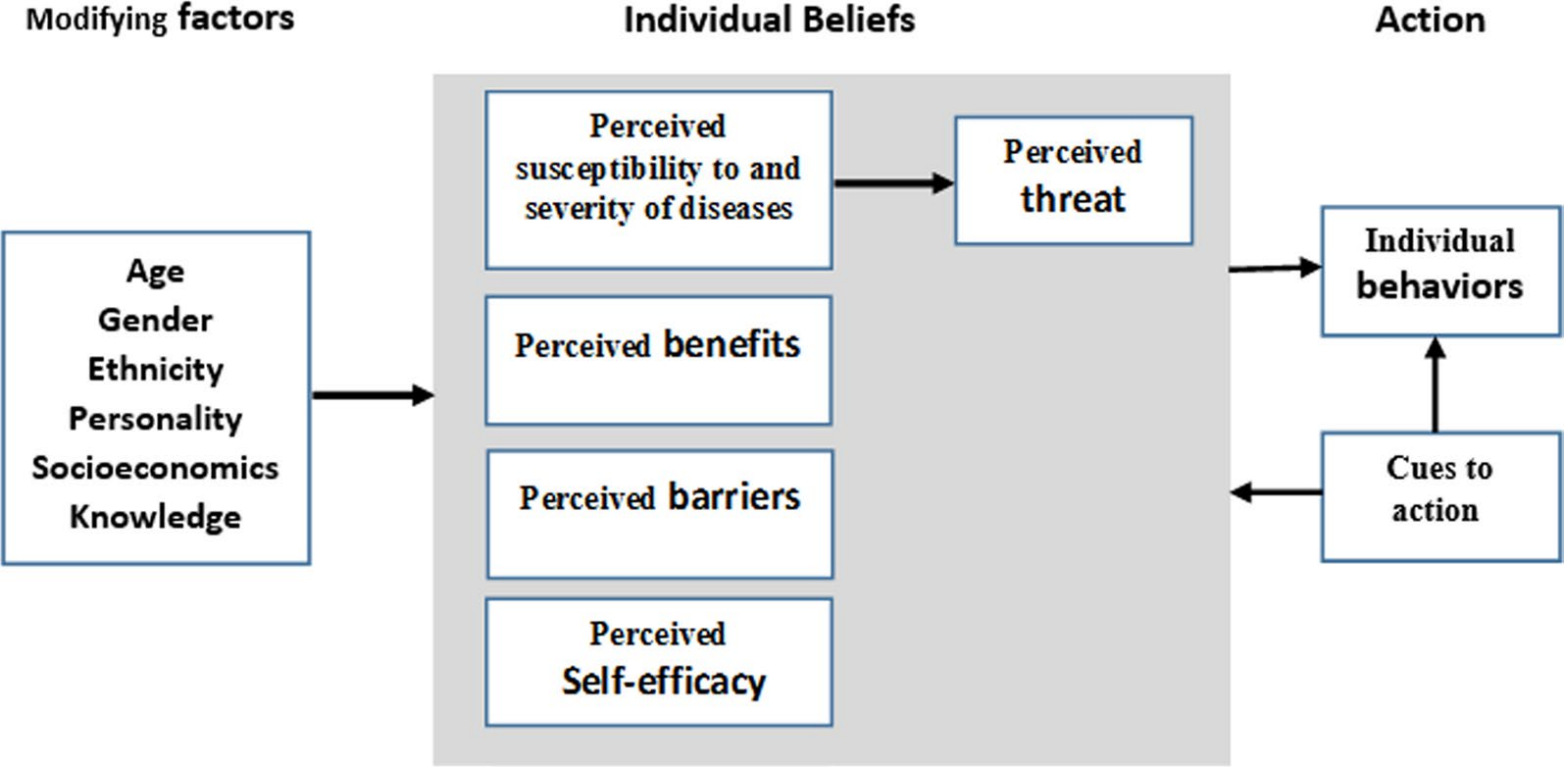
Components of the health belief model [21]

The Health Belief Model (HBM) developed in the 1950s by Godfrey Hochbaum, Irwin Rosenstock, and Rosenstock and Kirscht. The model constructs are perceived susceptibility and severity of diseases, perceived benefits of preventive behavior, perceived barriers to preventive behavior, cause to action and self-efficacy for doing preventive behavior. The definition of model constructs is as follows: "perceived susceptibility; beliefs about the likelihood of getting a disease or condition. Perceived severity; beliefs about the seriousness of contracting a diseases or condition, including consequences. Perceived benefits; beliefs about the positive aspects of adopting a health behavior (e.g., efficacy of the behavior for reducing risk or serious consequences). Perceived barriers; beliefs about the obstacles to performing a behavior, and the negative aspects (both tangible and psychological costs) of adopting a health behavior. Cues to action; internal and external factors that could trigger the health behavior. Self-efficacy; beliefs that one can perform the recommended health behavior" [21] (Fig. 1).

In their study, Khalilipour Darestani and Panahi reported that beliefs and perceptions of the adolescent's female about Premenstrual Syndrome (susceptibility, severity, benefits, barriers and perceived self-efficacy) in Tehran were moderate and lower than normal [22].

Given the role and importance of adolescent and young girls in society, the present study has been conducted to evaluate the role of health literacy and health beliefs in student's health promoting behaviors based on the Health Belief Model.

## Methods

### Study design and participants

The descriptive study has been conducted on 431 female students of Rafsanjan University of Medical Sciences selected by stratified sampling method in 2020. After determining the sample size by considering a 20% attrition using the formula n = Z^2^*P (1-P)/d^2^ [Z value is equal to 1.96 and P value is based on the study conducted by Saeedi Mottaghi and Koopaei [2] and is equal to 42% for optimal H.L, and the d value is equal to 0.05], the number of female students in each faculty was determined, and in proportion to their number, the required sample size was determined for each faculty. Then, by referring to the Education Department of each faculty, the link of the questionnaire created on Porsline was sent to as many female students as required. The data collection method was electronic by applying Porsline system.

### Data collection tools

Data collection tools included a questionnaire including an assessment of demographic information [age, academic year (being a freshman, sophomore, junior, and senior student), and level of education, being a local student, parents' educational level and occupation].

Health Literacy assessment questionnaire has been taken from the study conducted by Saeedi Kopaei and Mottaghi with 41 questions in five dimensions included menstrual, nutritional, physical activity, breast self-examination and anemia [2]. The dimensions of the questionnaire, the range of scores, validity and reliability and other features of this tool with the questions are provided in the Additional file 1.

In the third part, it was attempted to assess the students’ beliefs based on the constructs of the health belief model. This researcher-made questionnaire with 50 questions was designed based on the constructs of the health belief model. Perceived susceptibility and severity about anemia, breast cancer and genital infections and menstrual disorders, perceived benefits aimed at understanding the impact and benefits of exercise and healthy diet in reducing anemia, breast cancer and menstrual disorders, perceived barriers for students to exercise, follow a healthy diet, and engage in menstrual and personal hygiene behaviors, self-efficacy and confidence to perform the mentioned activities, cues to action the concept of students' understanding of the internal and external stimuli that motivate preventive behaviors. Full details of this section with questions are provided in the Additional file 1.

In this study, preventive behaviors of anemia, breast cancer, genital infections and menstrual disorders were evaluated with 9 questions such as regular exercise, healthy diet and personal hygiene and menstruation. Profile of the questions of this section is provided in the Additional file 1.

### Analysis of statistical data

The data were collected and analyzed by using SPSS V-20. Independent *t*-test to compare the mean score of a quantitative variable in two groups, one-way ANOVA to compare the mean score of a quantitative variable in several groups, Pearson correlation to determine the correlation between quantitative variables, and Multiple Linear Regression to determine the most important dependent variable predictors have been analyzed at the significance level less than of 0.05.

## Results

### Description of participants

The mean age of students was 23.50 ± 5.63 years ranging from 18 to 41 years. Around 61% of the students were undergraduate students. As many as 50% were freshmen and senior students, and 54% were non-local students. Moreover, about 70% of the students’ parents had a high school diploma or higher. As many as 50% of the students’ fathers were self-employed, and 31% of the students’ mothers were employed.

### Relationship between demographic characteristics and behavior

There was a direct and significant correlation between mean score and standard deviation (M ± SD) of behavior and age (r = 0.09, *p* = 0.042). However, one-way ANOVA test did not show a significant difference between the M ± SD of preventive behaviors and the students’ academic year (F = 0.71, *p* = 0.64).

One-way ANOVA and independent *t*-test did not show a significant difference between M ± SD of preventive behaviors in terms of different variables including father’s educational level (*p* = 0.19), mother’s educational level (*p* = 0.22), mother’s occupation (*p* = 0.91), and students’ being local (*p* = 0.10). Students whose father’s job was a “clerk” had the highest score of preventive behaviors (34.59 out of 100), and students whose fathers were “unemployed” had the lowest mean score of preventive behaviors (29.63 out of 100).

One-way ANOVA indicated that there is a significant difference between the M ± SD of preventive behavior based on the occupational status of the students’ fathers (*p* = 0.002). Post-hoc LSD indicated a significant difference between the M ± SD of a student’s preventive behaviors and his/her father’s job; construction worker vs. clerk (*p* = 0.037), clerk vs. unemployed (*p* = 0.001), construction worker vs. self-employed (*p* = 0.039), retired vs. unemployed (*p* = 0.001), unemployed vs. farmer (*p* = 0.032), and unemployed vs self-employed (*p* = 0.001) (Table 1).

**Table 1:** The mean and SD of students’ preventive behaviors in terms of their fathers’ job and educational grade

Variable	Status	Number	Mean ± SD	*p*-value
Father’s job	Employee	123	34.59 ± 5.47	0.002
	Worker	23	32.00 ± 5.98	
	Retired	125	34.19 ± 5.61	
	Unemployed	19	29.63 ± 4.41	
	Farmer	30	33.06 ± 5.19	
	Self-employed	111	34.58 ± 5.34	
	Sum	431	34.01 ± 5.53	
Educational grade	Associate	20	32.95 ± 5.94	0.048
	Bachelor	246	34.08 ± 5.41	
	master’s degree	62	35.45 ± 5.99	
	Ph.D. and doctorate	85	32.98 ± 5.33	
	Sum	431	5.53 ± 34.01	

Moreover, there was a significant difference between the M ± SD of students’ preventive behaviors in different academic levels (bachelor/master/Ph.D.) (*p* = 0.048). By conducting post-hoc LSD test, a significant difference was observed between the mean scores of preventive behaviors of master’s degree students and students of medicine and specialty (*p* = 0.008) (Table 1).

### Mean score and standard deviation of health literacy, students' beliefs and behavior

**Fig. 2:**
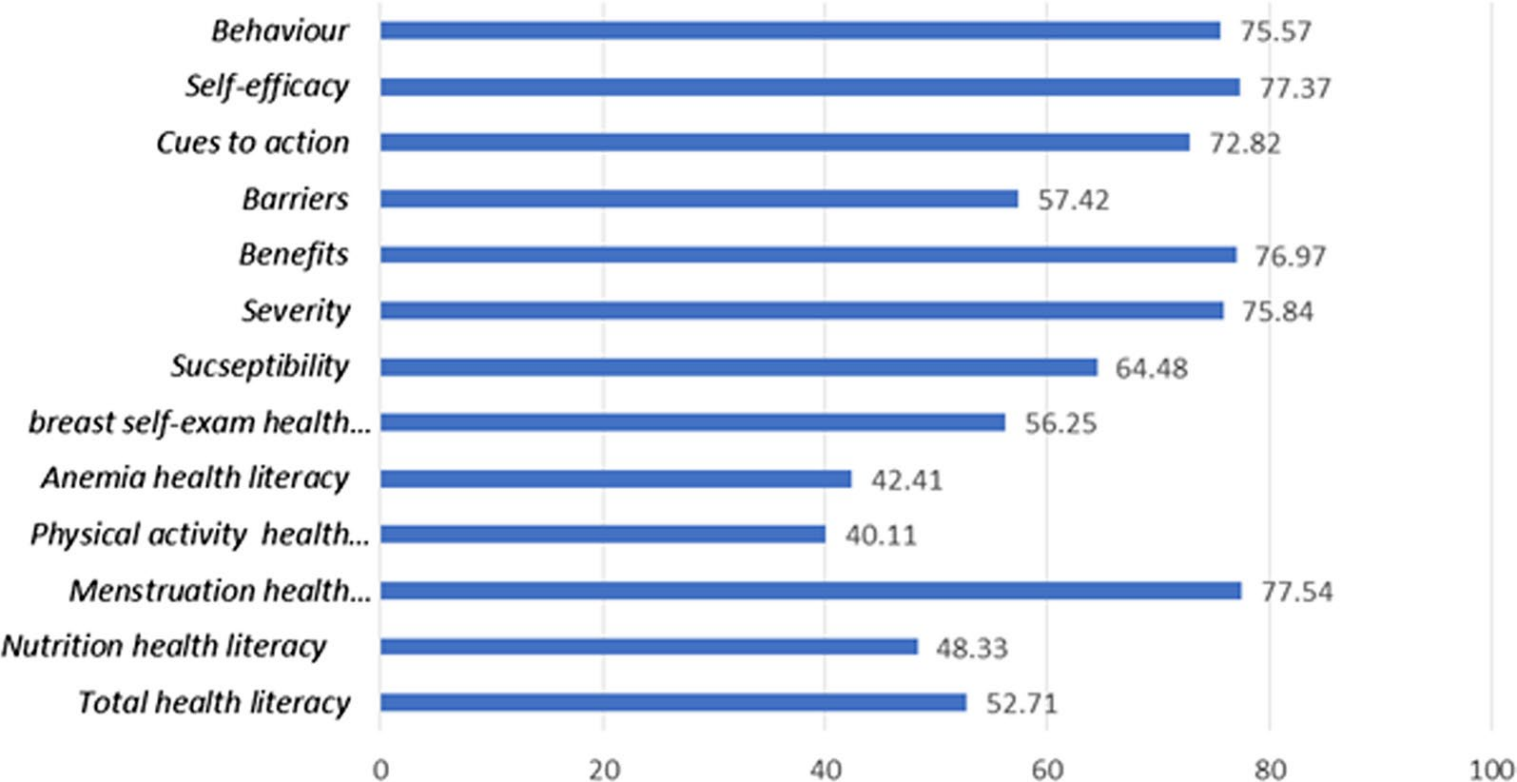
The scores of behavior, health literacy, and beliefs of female medical students about common diseases

Figure 2 shows the M ± SD of H.L and its dimensions i.e. students’ beliefs and preventive behaviors (Fig. 2). According to the graph, as many as 75.57% of students had adopted preventive health behaviors, and the total H.L score was 52.71 out of 100. The lowest percentage of H.L is related to Physical activity equal to 40.11%, and the highest percentage is related to menstruation being 77.54%. Moreover, the students’ highest score of beliefs was related to self-efficacy construct being 77.37%.

### Correlation between preventive behaviors with health literacy and students' beliefs

Table 2 shows the correlation between M ± SD of preventive behaviors with M ± SD of health beliefs and H.L and its dimensions (Table 2). The highest correlation was observed between behavior and total H.L (r = 0.414, *p* = 0.001), and as for the beliefs, the highest correlation was with self-efficacy (r = 0.642, *p* = 0.001). In this study, according multiple regression analysis, the strongest predictors of behaviors are in the following order: self-efficacy (β = 0.414, *p* = 0.001), cues to action (β = 0.299, *p* = 0.001), H.L (β = 0.170, *p* = 0.001), and perceived susceptibility (β = 0.099, *p* = 0.005). Thus, Health literacy, self-efficacy, cues to action and perceived susceptibility constructs predicted 52.1% of preventive behaviors (Table 3).

**Table 2:** Correlation of health beliefs and health literacy and its dimensions with preventive behaviors in female students

Variable Mean and SD	health literacy	Menstruation	Nutrition	Physical activity	Self-exam	Anemia	Susceptibility	Severity	Benefits	Benefits	Cues to action	Self-efficacy	Behavior
Health literacy 27.94 ± 6.96	1												
Menstruation 5.80 ± 2.00	0.67*	1											
Nutrition 8.53 ± 2.44	0.66*	0.36*	1										
Physical activity 4.01 ± 1.93	0.49*	0.15**	0.01^a^	1									
Self-exam 5.09 ± 2.35	0.74*	0.37*	0.29*	0.31*	1								
Anemia 4.50 ± 1.73	0.72*	0.38*	0.41*	0.25*	0.44*	1							
Susceptibility 32.24 ± 3.32	0.22*	0.23*	0.05^a^	0.18*	0.15*	0.13**	1						
Severity 49.30 ± 7.11	0.32*	0.22*	0.30*	0.10**	0.18*	0.23*	0.23*	1					
Benefits 26.94 ± 3.61	0.40*	0.27*	0.42*	0.14**	0.17*	0.31*	0.21*	0.51*	1				
Barriers 14.35 ± 3.42	− 0.30*	− 0.07^a^	− 0.32*	− 0.13**	− 0.19*	− 0.25*	− 0.01^a^	0.10**	− 0.18*	1			
Cues to action 29.13 ± 4.74	0.28*	0.15**	0.13**	0.26*	0.20*	0.22*	0.19*	0.32*	0.38*	− 0.05^a^	1		
Self-efficacy 27.08 ± 4.26	0.35*	0.17*	0.27*	0.25*	0.20*	0.25*	0.20*	0.36*	0.54*	− 0.239	0.53*	1	
Behavior 34.01 ± 5.53	0.41*	0.26*	0.25*	0.31*	0.27*	0.27*	0.26*	0.26*	0.38*	− 0.19*	0.57*	0.64*	1

*p < 0.001, **p < 0.01

^a^*p* > 0.05

**Table 3:** Multiple linear regression of perceptions and health literacy with preventive behaviors in female students

Variable and model	Non-standard coefficients		Standard coefficients	*t*-value	*p*-value	Summary
	B	SE	Beta			
Constant value	2.25	2.39	–	0.93	0.34	R = 0.427
Total health literacy	0.13	0.03	0.17	4.30	˂ 0.001	
Susceptibility	0.16	0.05	0.09	2.83	0.005	R^2^ = 0.528
Severity	− 0.05	0.03	− 0.07	− 1.80	0.07	
Benefits	0.04	0.06	0.02	0.61	0.53	Adjusted R^2^ = 0.521
Barriers	− 0.02	0.06	− 0.01	− 0.36	0.71	
Cues to action	0.34	0.04	0.29	7.33	˂ 0.001	Std. error of the estimate = 3.834
Self-efficacy	0.53	0.05	0.41	9.51	˂ 0.001	

*Dependent variable: preventive behavior

**Predicting variables (constant value): self-efficacy, cues to action, health literacy and susceptibility

## Discussion

In the present study, the H.L score was measured to be 52.71 out of 100. The highest H.L was related to menstruation and the lowest was related to physical activity. In Saeedi Kopaei’s study in Iran, Isfahan city, the total health literacy score of high school female students was 42.6 out of 100. H.L of menstrual health was 68.12 out of 100; it was 54.5% regarding breast self-examination, 48.5% regarding iron deficiency anemia, 81.23% about physical activity and 77.36% for nutrition H.L [2]. The lowest health literacy was related to anemia and breast self-examination and the highest score was related to physical activity. But in our study, the lowest score related to physical activity. The difference in this result can be attributed to the age of the participants (17.4 years vs. 23.5 years) and high school education versus university. Perhaps the heavy volume of lessons has reduced students' physical activity.

In the study conducted by Ahmadi et al. on female students, the H.L score was reported to be 67.28 out of 100 [23]. In another study conducted by Dehghankar et al. on female students, as many as 65.6% of the girls in the study had adequate and excellent H.L [24]. In a national study conducted on the general population in Iran, the mean H.L score was reported to be 69.02 in the general population of Iran [25]. The results of these studies also report moderate health literacy which is almost similar to the results of the present study.

Although the findings of the aforementioned study, in comparison to those our study, indicate that, contrary to what is expected, female college students of our study are less healthy than the general population, in another national study conducted by Haghdoost et al., H.L in the general population was 51%, and it was close to the findings of our study [26]. Different factors such as differences in populations investigated, sampling methods, and H.L assessment tools can result in differences in the findings of different studies. Moreover, in the study conducted by Tavousi [25] et al., it has been indicated that with increasing age (up to 44 years), H.L tends to increase; H.L was higher for people with the age range of 35–44 years those being in the age range of 18–24 years. This is likely to explain the difference between the findings of our study and those of the study conducted by Tavousi et al. [25].

In this study, there is a significant correlation between total H.L and health-promoting behaviors; when H.L increases, conducting these behaviors increases as well. Like our study, a correlation between H.L and health-promoting behaviors has been indicated in the study conducted (on 375 female college students of Imam Khomeini International University in Qazvin, Iran) by Panahi et al. as well [27]. The study by Mahdavi et al. conducted among 500 women who referred to family health unit in Tehran, Iran also confirmed the findings of our study on the correlation existing between H.L and preventive behaviors [28]. 48.6% of participants had low health literacy level, 24.4% had marginal level and only 27% had adequate health literacy level [28].

Therefore, it seems that promoting public H.L through mass media, social networks, and university curricula are likely to result in increased health-promoting behaviors. It is suggested that by designing, implementing and evaluating educational programs based on risk factors and reducing the burden of diseases in universities, serious attention be paid to the health of female students.

In addition, given the correlation between H.L and health-promoting behaviors and the existence of a significant relationship between health-promoting behaviors and age, educational level (bachelor, master …) and father’s job, it can be hypothesized that H.L has a significant relationship with these factors as well. This has been already indicated in previous studies [25–28]. Also, considering the presentation of similar results in several studies on the poor health literacy of Iranian women regarding diseases and health-promoting behaviors, the necessary planning should be done by the Legal Office of Women's Health in the country.

Based on the results of the present study, self-efficacy, cues to action, and perceived susceptibility are the strongest predictors of health-promoting behaviors. Similarly, in a study conducted by Kenari et al. (on school students in Rasht), perceived self-efficacy was identified as the most important predictor of health behaviors. In the study conducted by Kenari et al., the cues to action and benefits were recognized to be the next important predictors [29]. In confirming our results, a study conducted by Ahmadian et al. (on students in Malaysia) indicated that perceived self-efficacy was the most important predictive factor of behavior. Moreover, in the aforementioned study, perceived barriers have been reported to be a negative factor in predicting behaviors [30]. Given the correlation between health belief model and health-promoting behaviors, some studies have indicated that a health belief model-based education is likely to increase health-promoting behaviors. For example, in a study on pre-university girls in Tehran, it was indicated that perceived self-efficacy increased significantly after providing education which was based on model. This education was proved to be effective on preventive behaviors [22]. In a study conducted by Karimi et al. (on nutritional behaviors of pregnant women), perceived benefits were recognized to have the highest correlation. After perceived benefits, it was shown that perceived barriers, susceptibility, severity, and self-efficacy were then correlated with nutritional behaviors [31]. The type of health-promoting behavior seems to be correlated with different components of the health belief model. For example, although in our study, perceived self-efficacy (as a whole) was most correlated with health-promoting behaviors, it was also partly indicated that having healthy diet, as a health-promoting behavior, is more correlated with perceived benefits, according to the results of Table Two. This has been indicated in the study conducted by Karimi et al. as well [31]. Also, physical activity behavior is more correlated with cues to action. Thus, these differences need to be attributed to differences in the type of behaviors. In a study conducted by Shirzad et al. (on girls living in child care centers in Tehran), it was shown that perceived susceptibility, benefits, and barriers are the most important predictive factors of health-promoting behaviors [32]. These differences are possibly owing to differences in social status of the statistical population in comparison to the population of our study.

Electronic response to the questionnaire has both weaknesses and strengths. Its strengths include faster data collection, ensuring that the data is confidential and anonymous, and providing honest answers due to the absence of the researcher. However, in the absence of the researcher, they may ask their friends the answers to some questions or search other sources at the time of completion.

Moreover, one of its strengths is the application of health belief model to assess students’ beliefs and perceptions. Generally speaking, it is suggested that intervention programs based on behavior change theories (such as health belief model and self-efficacy theory) be considered by the related university officials with the aim of promoting students’ H.L and self-efficacy to perform health-promoting behaviors for female students. Because by promoting students' beliefs (perceived susceptibility and severity) about diseases and increasing the benefits and removing barriers to health-promoting behaviors, in addition to increasing self-efficacy, their health literacy will also increase and as a result behavior will change.

## Conclusion

Approximately 75% of the students had adopted preventive health behaviors for women’s diseases. The total H.L score was 52.71 out of 100. The lowest percentage of H.L was related to physical activity being 40.11%, and the highest score was related to menstruation being 77.54%. Moreover, the highest score of students’ beliefs was related to self-efficacy construct being 77.37%. The highest correlation was observed between behavior and total H.L (r = 0.414, *p* < 0.0001), and as for the beliefs, the highest correlation was with self-efficacy (r = 0.642, *p* < 0.0001). In this study, according multiple regression analysis, the strongest predictors of behaviors are self-efficacy (Beta = 0.414, *p* < 0.001) and cues to action (Beta = 0.299, *p* < 0.001). Thus, Health literacy, self-efficacy, cues to action and perceived susceptibility constructs predicted 52.1% of preventive behaviors.

## Supplementary Information

**Additional file 1.** Specifications of the questionnaire used in the research.

## Data Availability

The data used and analyzed during the current study available from the corresponding author.

## Abbreviations

HL: Health literacy
HBM: Health belief model
