# Supplementary material for: The role of health beliefs and health literacy in women's health promoting behaviours based on the health belief model: a descriptive study
Source: BMC Womens Health. 2021 Dec 18;21:421. doi: 10.1186/s12905-021-01564-2 (PMC8684276; doi:10.1186/s12905-021-01564-2)
Supplement: Supplementary file 1 — Additional file 1. Specifications of the questionnaire used in the research. [file 12905_2021_1564_MOESM1_ESM.pdf]

Supplementary file: specifications of the questionnaire used in the research

The Role of Health Beliefs and Health Literacy in Women's Health Promoting Behaviours based on the Health Belief Model: A descriptive study

Section-1: health literacy (H.L)

This questionnaire with 41 items evaluates students' health literacy regarding menstrual health (questions 1 to 8), nutrition (questions 9 to 19), physical activity (questions 20 to 25), breast cancer diagnostic self-examination (questions 26 to 35) and iron deficiency (Questions 36 to 41). The scale of answering questions is 4 options that are given to the correct answer of grade two, the wrong answer of grade zero and the answer I do not know grade one. The range of grades is between zero and 82, which higher score indicates the higher health literacy of students in the above subjects. Scores were calculated out of 100.

Scores in each subscale (subject) will be calculated and the score of each section and the total score of the questionnaire will be divided into three divisions and three limited general categories: low health literacy score, moderate health literacy score and optimal health literacy score. For example, the total range of the questionnaire is between zero and 82, obtaining a score between zero and 27 is classified as unfavorable health literacy, a score of 27 to 54 as moderate health literacy, and a score of 54 to 82 as classified as good health literacy.

How to respond is self-reporting. This questionnaire is taken from the research of Saeedi-Koupai and Motaghi (2). In their research, it has been reported that the validity of the instrument has been reviewed and confirmed by ten masters of the Faculty of Nursing and Midwifery of Khorasgan Azad University of Isfahan with the help of content validity index and content validity ratio. Its reliability was evaluated in a pilot study by 20 students and its Cronbach's alpha coefficient was reported to be 0.7.

Also in the present study, after students answered the questionnaire, Cronbach's alpha coefficient of health literacy questions was evaluated which was equal to 0.79.

Health Literacy Questionnaire

1. What method do you use to reduce menstrual pain?
   A. Home remedies ○
   B. Taking painkillers ○
   C. Refer to Medical Center ○
   D. I do not know ○

2. How many times do you use a hot shower during your period?
   A. Every day ○
   B. Once every two days ○
   C. Not at all ○
   D. I do not know ○

3. During menstruation after washing the genital area
   A. The genital area should not be dried ○
   B. This area should be completely dry ○
   C. A clean and non-contaminated sanitary napkin should be used. ○
   D. I do not know ○

4. Which of the following is the best way to prevent unpleasant odors during menstrual cycle?
   A. Washing the genital area with aromatic soaps ○
   B. Use lukewarm water to wash ○
   C. Use of aromatic sanitary napkins ○
   D. I do not know ○

5. Which option is correct about covering the genital area during menstrual cycle?
   A. Use plastic underwear because these clothes get infected late. ○
   B. Use underwear made of cotton ○
   C. In this period, the role of underwear is not important and only much attention should be paid to the quality of the sanitary napkin. ○
   D. I do not know ○

6. What should be the form of washing the genital area during menstrual cycle?
   A. Washing and drying should be done from the anus to the front. ○
   B. Washing and drying should be done from the front to the anus. ○
   C. The direction of washing is not important and what is important is washing that area. ○
   D. I do not know ○

7. Which option is wrong to reduce menstrual pain?
   A. Use of hot shower ○
   B. Stop consuming caffeine-containing products, such as coffee. ○
   C. Not taking painkillers ○
   D. I do not know. ○

8. Which option do you think is right about menstrual cycle?
   A. Try to rest more during this period. ○
   B. Any physical activity can cause sweating and infection of the genital area. ○
   C. Physical activity and light exercise can relax a person. ○
   D. I do not know. ○

9. Which option is a natural laxative and is used to prevent constipation?

A. Fruits and vegetables ○
B. Wholemeal flour ○
C. Edible oils ○
D. I do not know ○

10. Omega-3 is effective in preventing premenstrual depression and Alzheimer's disease, etc. Which of the following sources is richer in omega-3?
    A. Fruits and vegetables ○
    B. Red meat ○
    C. Salmon ○
    D. I do not know ○

11. Which food has an unlimited shelf life?
    A. Flour ○
    B. Sugar ○
    C. Salt ○
    D. I do not know. ○

12. What is the best way to prevent osteoporosis in women?
    A. Getting enough calcium and vitamin D with exercise. ○
    B. Getting calcium and sun exposure ○
    C. Regular visits to relevant specialists ○
    D. I do not know ○

13. Which of the following foods does not contain cholesterol?
    A. Solid oils ○
    B. Egg yolk ○
    C. Fish ○
    D. I do not know ○

14. What is the best and healthiest diet?
    A. A diet that contains enough nutrients ○
    B. A diet that contains different types of nutrients in the four main food groups ○
    C. A diet that contains sufficient amounts of different types of food in the four main food groups ○
    D. I do not know ○

15. Which vitamins do lemon, orange and tomato juices often contain?
    A. Vitamin A ○
    B. Vitamin B ○
    C. Vitamin C ○
    D. I do not know ○

16. Dairy products such as milk and yogurt provide what substances the body needs?
   A. Cholesterol ○
   B. Calcium ○
   C. Vitamin A ○
   D. I do not know ○

17. What is the most important cause of osteoporosis?
   A. Lack of exercise and mobility ○
   B. Use of canned food ○
   C. Do not use foods containing cholesterol ○
   D. I do not know ○

18. What is the most common cause of anemia in women?
   A. Deficiency of fat in the body ○
   B. Iron deficiency ○
   C. Immobility and exercise ○
   D. I do not know ○

19. Lentils and other legumes are more useful and effective in providing which of the following sources?
   A. Vitamin A ○
   B. Iron ○
   C. Cholesterol ○
   D. I do not know ○

20. How many times a week do you exercise?
   A. Less than 3 days ○
   B. 3-6 days ○
   C. 7 days ○
   D. I do not know ○

21. How much physical activity is recommended for young people during a week?
   A. Three sessions or more each week for at least 20 minutes (Moderate to high--intensity activity) ○
   B. One session per week for an hour of moderate-intensity activity ○
   C. Two sessions per week for two hours of low-intensity activity ○

22. Is climbing stairs harmful for healthy people?
   A. Yes, it causes early wear and tear on a person's joint surfaces. ○
   B. No. It strengthens the joints and strengthens the muscles around the joints. ○
   C. No effect ○
   D. I do not know ○

23. Which option is wrong about exercise and physical activity?
   A. Exercise delays the phenomenon of aging. ○
   B. Exercise prevents women from getting breast cancer. ○
   C. Exercise promotes fitness but has no effect on the incidence and prevention of disease. ○
   D. I do not know ○

24. How many days a week do you walk at least 10 minutes continuously?
   A. Less than 3 days ○
   B. 3-6 days ○
   C. 7 days ○
   D. I do not know ○

25. When does exercise help your fitness?
   A. Morning ○
   B. Afternoon ○
   C. J. Nigh ○
   D. I do not know ○

26. At what age should breast self-examinationination begin?
   A. 16 ○
   B. 18 ○
   C. 20 ○
   D. I do not know ○

27. Which of the following is true about breast self-examination?
   A. Breast self-examination should be done daily. ○
   B. The best time to do a breast self-examination is before menstruation begins ○
   C. The best time to do a breast self-examination is after a bath after your menstrual cycle ends. ○
   D. I do not know. ○

28. Which of the following is not a dangerous sign in the chest?
A. Sunken skin or nipple ○
B. New change in breast size ○
C. Breast asymmetry ○
D. I do not know ○

29. For proper breast self-examination.
A. Fingers should be used. ○
B. The palms of the hands should be used. ○
C. The best method is self-eye examination. ○

D. I do not know ○

30. At what age should mammography be started?
A. 20 ○
B. 30 ○
C. 40 ○
D. I do not know. ○

31. Which foods do you think increase the risk of breast cancer
A. Fatty and salty foods ○
B. Canned foods ○
C. Foods cooked in the microwave ○
D. I do not know. ○

32. What foods are effective in preventing breast cancer?
A. Vegetables and fruits ○
B. Foods smoked ○
C. Toasted breads ○
D. I do not know. ○

33. Which does not prevent breast cancer?
A. Use of foods containing omega 3 ○
B. Use of soy and its products ○
C. Use of foods containing iron ○
D. I do not know. ○

34. Which option do you think is wrong about the factors affecting the incidence of breast cancer?
A. Pipe cigarettes and hookah are among the factors that increase breast cancer. ○
B. Stress has little effect on breast cancer. ○
C. 30 minutes of exercise a day reduces the risk of breast cancer in women. ○
D. I do not know. ○

35. To what extent do you consult a relevant specialist about breast abnormalities?
A. Rarely ○
B. Much ○
C. Very much ○
D. I do not know. ○

36. Which vitamin deficiency in the body causes anemia more than other cases?
A. Vitamin A ○
B. Vitamin B12 ○
C. Vitamin D ○
D. I do not know. ○

37. Which option is not correct about taking iron pills?

A. Use fruits and vegetables to prevent constipation caused by iron tablets. ○

B. To reduce nausea and vomiting caused by taking iron tablets, it can be taken after a meal. ○

C. If you have abdominal pain and dark stools after taking iron tablets, you should stop taking them and see a doctor. ○

D. I do not know. ○

38. Which of the following is true about you?

A. I rarely eat red meat a week. ○

B. I usually drink tea after eating. ○

C. I'm not used to using vegetables ○

D. I do not know. ○

39. Which option is not correct?

A. Anemia in adolescence can reduce growth rate, reduce IQ and reduce learning power. ○

B. Use sprouted grains and legumes in your daily diet to prevent iron deficiency and anemia. ○

C. Consumption of herbal infusions has no effect on the incidence of iron deficiency anemia. ○

D. I do not know. ○

40. What is the best way to diagnose anemia?

A. Cooling of hands and feet ○

B. Menopause ○

C. Performing blood test ○

D. I do not know. ○

41. What do you think is the best way to absorb iron from food?

A. Drinking water at the beginning and end of food ○

B. Eating iron tablets ○

C. Eating citrus fruits and vegetables with food or between two meals. ○

D. I do not know. ○

Section 2: Researcher-made questionnaire about the students' beliefs based on the constructs of the health belief model

This questionnaire assesses students' beliefs about anemia, breast cancer, and genital infections and menstrual disorders with a focus on preventative behaviors such as exercise, a healthy diet, and personal hygiene and menstruation.

The questions are consistent with the constructs of the health belief model developed by the research team. Its validity (CVI, CVR) was evaluated with the help of ten specialists in midwifery and nursing and health education and health promotion. Reliability in a pilot study was assessed by 17 students and the final questionnaire was presented after modification.

Perceived susceptibility:

This construct assessed students' susceptibility to getting probability of anemia, breast cancer, genital infections, and menstrual irregularities with 10 questions. The response scale with Likert was 5 options from strongly agree to strongly disagree. A score of five was given to the answer "strongly agree" and a score of one was given to the answer "strongly disagree". The scores in this section ranged from 10 to 50. A higher score indicated the susceptibility of students' higher understanding of the likelihood of getting these diseases.

1. I believe I am at risk for iron deficiency anemia due to my current physical condition.
   A. Strongly agree ○
   B. Agree ○
   C. Neither agree nor disagree ○
   D. Disagree ○
   E. Strongly disagree ○

2. I believe I am not at risk of developing iron deficiency anemia due to proper nutrition and adherence to healthy eating principles.
   A. Strongly agree ○
   B. Agree ○
   C. Neither agree nor disagree ○
   D. Disagree ○
   E. Strongly disagree ○

3. I believe I will not be at risk for iron deficiency anemia with regular exercise (3-5 times a week and at least 30 minutes each time).
   A. Strongly agree ○
   B. Agree ○
   C. Neither agree nor disagree ○
   D. Disagree ○
   E. Strongly disagree ○

4. I believe I am also at risk for breast cancer due to the prevalence of breast cancer in the family or family history.
   A. Strongly agree ○

B. Agree ○
C. Neither agree nor disagree ○
D. Disagree ○
E. Strongly disagree ○

5. I believe I am less at risk for breast cancer due to my healthy eating style (such as regular consumption of fruits and vegetables and not eating fast foods).
   A. Strongly agree ○
   B. Agree ○
   C. Neither agree nor disagree ○
   D. Disagree ○
   E. Strongly disagree ○

6. I believe I will be more at risk for cancer because I do not exercise.
   A. Strongly agree ○
   B. Agree ○
   C. Neither agree nor disagree ○
   D. Disagree ○
   E. Strongly disagree ○

7. I believe I am at risk for breast cancer.
   A. Strongly agree ○
   B. Agree ○
   C. Neither agree nor disagree ○
   D. Disagree ○
   E. Strongly disagree ○

8. I believe I am at risk for genital infections because I do not care about full compliance with health tips, including personal hygiene and genital area.
   A. Strongly agree ○
   B. Agree ○
   C. Neither agree nor disagree ○
   D. Disagree ○
   E. Strongly disagree ○

9. I believe that menstrual pain will be reduced by observing the principles of healthy and appropriate food (such as regular consumption of dairy products, fruits and vegetables and not consuming fast foods).
   A. Strongly agree ○
   B. Agree ○
   C. Neither agree nor disagree ○
   D. Disagree ○
   E. Strongly disagree ○

10. I believe that observing personal health behaviors during menstruation does not have a significant effect on reducing menstrual pain.
    A. Strongly agree ○
    B. Agree ○
    C. Neither agree nor disagree ○
    D. Disagree ○
    E. Strongly disagree ○

Perceived severity:

With 13 questions, the construct assessed students' perceived severity of anemia, breast cancer, genital infections, and menstrual irregularities. What are the consequences for me if I suffer from the above diseases? The response scale with Likert was 5 options from strongly agree to strongly disagree. A score of five was given to the answer "strongly agree" and a score of one was given to the answer "strongly disagree". The scores in this section ranged from 13 to 65. A higher score indicated the perceived severity of students' higher of other complications and possible consequences of the disease.

1. I believe that iron deficiency anemia is one of the most important and serious diseases of women.
    A. Strongly agree ○
    B. Agree ○
    C. Neither agree nor disagree ○
    D. Disagree ○
    E. Strongly disagree ○

2. I believe that having iron deficiency anemia causes mental disorders such as depression.
    A. Strongly agree ○
    B. Agree ○
    C. Neither agree nor disagree ○
    D. Disagree ○
    E. Strongly disagree ○

3. I believe that iron deficiency anemia causes fatigue and boredom.
    A. Strongly agree ○
    B. Agree ○
    C. Neither agree nor disagree ○
    D. Disagree ○
    E. Strongly disagree ○

4. I believe that having iron deficiency anemia causes delays in daily activities.
   A. Strongly agree ○
   B. Agree ○
   C. Neither agree nor disagree ○
   D. Disagree ○
   E. Strongly disagree ○

5. I believe that breast cancer is one of the most serious and dangerous diseases in women.
   A. Strongly agree ○
   B. Agree ○
   C. Neither agree nor disagree ○
   D. Disagree ○
   E. Strongly disagree ○

6. I believe that breast cancer causes mental disorders such as depression.
   A. Strongly agree ○
   B. Agree ○
   C. Neither agree nor disagree ○
   D. Disagree ○
   E. Strongly disagree ○

7. I believe that breast cancer causes disruption in social relationships such as the loss of my friends.
   A. Strongly agree ○
   B. Agree ○
   C. Neither agree nor disagree ○
   D. Disagree ○
   E. Strongly disagree ○

8. I believe that breast cancer can cause a lot of stress for my family.
   A. Strongly agree ○
   B. Agree ○
   C. Neither agree nor disagree ○
   D. Disagree ○
   E. Strongly disagree ○

9. I believe that breast cancer causes serious economic problems.
   A. Strongly agree ○
   B. Agree ○
   C. Neither agree nor disagree ○
   D. Disagree ○
   E. Strongly disagree ○

10. I believe it will be very difficult for me to continue living if I get breast cancer.
    A. Strongly agree ○
    B. Agree ○
    C. Neither agree nor disagree ○
    D. Disagree ○
    E. Strongly disagree ○

11. I believe I will suffer from mental disorders if I suffer from infections and diseases of the genital area.
    A. Strongly agree ○
    B. Agree ○
    C. Neither agree nor disagree ○
    D. Disagree ○
    E. Strongly disagree ○

12. I believe that having infections and diseases of the genital area will disrupt my social relationships and even have a serious impact on my relationship with my friends.
    A. Strongly agree ○
    B. Agree ○
    C. Neither agree nor disagree ○
    D. Disagree ○
    E. Strongly disagree ○

13. I believe my family will suffer from mental disorders if they suffer from infections and diseases of the genital area.
    A. Strongly agree ○
    B. Agree ○
    C. Neither agree nor disagree ○
    D. Disagree ○
    E. Strongly disagree ○

Perceived benefits:

With 7 questions, this construct evaluated the perceived benefits of students regarding the effects and benefits of exercise, healthy diet and personal hygiene and menstruation on the prevention of anemia, breast cancer and infections and menstrual disorders. The response scale with Likert was 5 options from strongly agree to strongly disagree. A score of five was given to the answer "strongly agree" and a score of one was given to the answer "strongly disagree". The scores in this section ranged from 7 to 35.

1. I believe that following the principles of healthy eating has a significant effect on preventing iron deficiency anemia.

A. Strongly agree ○
B. Agree ○
C. Neither agree nor disagree ○
D. Disagree ○
E. Strongly disagree ○

2. I believe that exercise has a significant effect on the prevention of iron deficiency anemia.
   A. Strongly agree ○
   B. Agree ○
   C. Neither agree nor disagree ○
   D. Disagree ○
   E. Strongly disagree ○

3. I believe that following the principles of healthy eating does not have a significant effect on the prevention of breast cancer.
   A. Strongly agree ○
   B. Agree ○
   C. Neither agree nor disagree ○
   D. Disagree ○
   E. Strongly disagree ○

4. I believe that exercising helps prevent breast cancer.
   A. Strongly agree ○
   B. Agree ○
   C. Neither agree nor disagree ○
   D. Disagree ○
   E. Strongly disagree ○

5. I believe that full observance of hygienic points, including personal hygiene and the genital area can prevent the occurrence of genital infections.
   A. Strongly agree ○
   B. Agree ○
   C. Neither agree nor disagree ○
   D. Disagree ○
   E. Strongly disagree ○

6. I believe that exercising, especially during menstrual cycle, will reduce menstrual pain.
   A. Strongly agree ○
   B. Agree ○
   C. Neither agree nor disagree ○
   D. Disagree ○
   E. Strongly disagree ○

7. I believe that paying attention to the principles of healthy eating during menstrual cycle will greatly reduce menstrual pain.
   A. Strongly agree ○
   B. Agree ○
   C. Neither agree nor disagree ○
   D. Disagree ○
   E. Strongly disagree ○

Perceived barriers:

This construct evaluated the perceived barriers for students to exercise regularly, have a healthy diet, and observe personal hygiene and menstruation with 5 questions. The response scale with Likert was 5 options from strongly agree to strongly disagree. A score of five was given to the answer "strongly agree" and a score of one was given to the answer "strongly disagree". The scores in this section ranged from 5 to 25. A higher score is the most perceived barrier for students to perform these behaviors.

1. I do not have enough time and opportunity to exercise.
   A. Strongly agree ○
   B. Agree ○
   C. Neither agree nor disagree ○
   D. Disagree ○
   E. Strongly disagree ○

2. I do not have enough information and knowledge about the principles of healthy eating with the aim of preventing iron deficiency anemia and breast cancer and promoting my own health.
   A. Strongly agree ○
   B. Agree ○
   C. Neither agree nor disagree ○
   D. Disagree ○
   E. Strongly disagree ○

3. I do not have enough money to buy healthy and nutritious foods to prevent iron deficiency anemia and breast cancer and improve my health.
   A. Strongly agree ○
   B. Agree ○
   C. Neither agree nor disagree ○
   D. Disagree ○
   E. Strongly disagree ○

4. It is difficult for me to follow a healthy diet due to my personality traits.
   A. Strongly agree ○

B. Agree ○
C. Neither agree nor disagree ○
D. Disagree ○
E. Strongly disagree ○

5. I do not believe in personal hygiene to prevent genital infections.
   A. Strongly agree ○
   B. Agree ○
   C. Neither agree nor disagree ○
   D. Disagree ○
   E. Strongly disagree ○

Cues to action

With 8 questions, this construct assessed students' understanding of internal and external stimuli that encourages them to perform regular exercise, have a healthy diet, and observe personal hygiene and menstruation in order to reduce the risk of disease. The response scale with Likert was 5 options from strongly agree to strongly disagree. A score of five was given to the answer "strongly agree" and a score of one was given to the answer "strongly disagree". The scores in this section ranged from 8 to 40.

1. My family encourages me to do proper exercise and physical activity.
   A. Strongly agree ○
   B. Agree ○
   C. Neither agree nor disagree ○
   D. Disagree ○
   E. Strongly disagree ○

2. My family supports me to eat healthy food and adhere the principles of healthy eating.
   A. Strongly agree ○
   B. Agree ○
   C. Neither agree nor disagree ○
   D. Disagree ○
   E. Strongly disagree ○

3. My friends encourage me to do proper exercise and physical activity.
   A. Strongly agree ○
   B. Agree ○
   C. Neither agree nor disagree ○
   D. Disagree ○
   E. Strongly disagree ○

4. My family supports me to eat healthy food and adhere the principles of healthy eating.
   A. Strongly agree ○
   B. Agree ○
   C. Neither agree nor disagree ○
   D. Disagree ○
   E. Strongly disagree ○

5. Television programs encourage me to do proper exercise and physical activity.
   A. Strongly agree ○
   B. Agree ○
   C. Neither agree nor disagree ○
   D. Disagree ○
   E. Strongly disagree ○

6. Television programs support me to eat healthy food and adhere the principles of healthy eating.
   A. Strongly agree ○
   B. Agree ○
   C. Neither agree nor disagree ○
   D. Disagree ○
   E. Strongly disagree ○

7. Recommendations of doctors and health experts encourage me to do proper exercise and physical activity.
   A. Strongly agree ○
   B. Agree ○
   C. Neither agree nor disagree ○
   D. Disagree ○
   E. Strongly disagree ○

8. Recommendations of doctors and health experts support me to eat healthy food and adhere the principles of healthy eating.
   A. Strongly agree ○
   B. Agree ○
   C. Neither agree nor disagree ○
   D. Disagree ○
   E. Strongly disagree ○

Perceived self-efficacy

With 7 questions, this construct assessed students' understanding of their confidence to perform regular exercise, have a healthy diet, and observe personal hygiene and menstruation. The response scale with Likert was 5 options from strongly agree to strongly disagree. A score of five was given to the answer "strongly agree" and a score of one was given to the answer "strongly disagree". The scores in this section ranged from 7 to 35.

1. I am sure I can exercise regularly (3-5 times a week and at least 30 minutes each time).
   A. Strongly agree ○
   B. Agree ○
   C. Neither agree nor disagree ○
   D. Disagree ○
   E. Strongly disagree ○

2. I am sure I can follow the principles of healthy eating.
   A. Strongly agree ○
   B. Agree ○
   C. Neither agree nor disagree ○
   D. Disagree ○
   E. Strongly disagree ○

3. I am sure I can observe my personal hygiene properly.
   A. Strongly agree ○
   B. Agree ○
   C. Neither agree nor disagree ○
   D. Disagree ○
   E. Strongly disagree ○

4. I am sure I can do proper health behaviors during menstrual cycle.
   A. Strongly agree ○
   B. Agree ○
   C. Neither agree nor disagree ○
   D. Disagree ○
   E. Strongly disagree ○

5. I'm sure I can pay attention to my family's helpful advice on exercising.
   A. Strongly agree ○
   B. Agree ○
   C. Neither agree nor disagree ○
   D. Disagree ○
   E. Strongly disagree ○

6. I am sure I can get the necessary information about health issues.
   A. Strongly agree ○
   B. Agree ○

C. Neither agree nor disagree ○
D. Disagree ○
E. Strongly disagree ○

7. I'm sure I can pay attention to my family's helpful advice on eating healthy foods like dairy.
A. Strongly agree ○
B. Agree ○
C. Neither agree nor disagree ○
D. Disagree ○
E. Strongly disagree ○

Validity and reliability of the tool

Validity

The expert panel method was used to determine the validity of the content qualitatively. The questionnaire was given to ten health education and health promotion and midwifery specialists. Their comments were received and included in the questionnaire. To determine the validity of the content quantitatively, the forms related to assess the content validity index and the content validity ratio were provided to the above experts, which are detailed in Table 1.

Table 1 the process of changing questions from the beginning of the design to the end of determining validity:

Constructs	Number of questions before validity	Number of questions after validity	Number of questions deleted (with experts 'opinion)	Number of questions added (with experts' opinion)	CVI	CVR
Perceived susceptibility	11	10	1	0	0.88	0.87
Perceived severity	11	13	0	2	0.93	0.90
Perceived benefits	7	7	0	0	0.92	0.87
Perceived barriers	6	5	1	0	0.97	0.89
Cues to action	8	8	0	0	0.88	0.84
Self- efficacy	8	7	1	0	0.98	0.86

Face validity was also assessed from the perspectives of 17 students, who were asked to rate the importance of each question on a 5-point Likert scale from "not important at all" to "absolutely important". In this section, the option does not matter at all was given a score of one and a very important score of 5 points, and the impact factor of each question in the structures was calculated using the product of the frequency of the percentage response in

importance, considering that all questions have a mean score higher than 1.5, do not delete the question in this section.

Reliability

The reliability or stability of the questionnaire was assessed using the test-retest method. In a pilot study, a questionnaire was given to 17 students (outside the study group) and then 14 days later the same questionnaire was given to them and the correlation coefficient of the answers was evaluated.

The correlation coefficient of all structures was above 0.9 and within the acceptable range. Also, due to the limitations of this method in evaluating attitude variables, Cronbach's alpha coefficient was measured; The Cronbach's alpha coefficient for the constructs of perceived susceptibility (0.78), perceived severity (0.85), perceived benefits (0.72), perceived barriers (0.68), self-efficacy (0.86), Cues to action (0.80) were calculated.

Section 3: Preventive behaviors

This construct was evaluated with 9 questions, the scale of which was measured by Likert 5 options from strongly agree to strongly disagree. A score of five was given to the answer "strongly agree" and a score of one was given to the answer "strongly disagree". The scores in this section ranged from 9 to 45. In this study, preventive behaviors of anemia, breast cancer, genital infections and menstrual disorders were evaluated with 9 questions such as regular exercise, healthy diet and personal hygiene and menstruation.

To perform the validity and reliability of this structure, all the above steps were performed, and finally a question was added based on the opinion of experts. The content validity index was 0.98, the content validity ratio was 0.86 and Cronbach's alpha coefficient was 0.82.

1. I exercise regularly (3-5 times a week and at least 30 minutes each time).
   A. Always ○
   B. Often ○
   C. Occasionally ○
   D. Rarely ○
   E. Not at all ○

2. I follow the principles and healthy diet such as regular consumption of fruits and vegetables and reducing the consumption of fast foods.
   A. Always ○
   B. Often ○
   C. Occasionally ○
   D. Rarely ○
   E. Not at all ○

3. I observe my personal hygiene properly.

A. Always ○

B. Often ○
C. Occasionally ○
D. Rarely ○
E. Not at all ○

4. I do proper health behaviors during menstrual cycle.
   A. Always ○
   B. Often ○
   C. Occasionally ○
   D. Rarely ○
   E. Not at all ○

5. I pay attention to my family's advice to take preventive behaviors such as eating fruits and vegetables and exercising.
   A. Always ○
   B. Often ○
   C. Occasionally ○
   D. Rarely ○
   E. Not at all ○

6. I pay attention to the recommendations of doctors and healthcare experts.
   A. Always ○
   B. Often ○
   C. Occasionally ○
   D. Rarely ○
   E. Not at all ○

7. I pay attention to my friends' advice for preventive behaviors such as eating fruits and vegetables and exercising.
   A. Always ○
   B. Often ○
   C. Occasionally ○
   D. Rarely ○
   E. Not at all ○

8. I take medication supplements such as multivitamins and iron regularly and regularly, according to the instructions.
   A. Always ○
   B. Often ○
   C. Occasionally ○
   D. Rarely ○
   E. Not at all ○

9. I am looking for information to improve my knowledge.

A. Always ○
B. Often ○
C. Occasionally ○
D. Rarely ○
E. Not at all ○
